# The Effect of Green Tea Ingestion and Interval Sprinting Exercise on the Body Composition of Overweight Males: A Randomized Trial

**DOI:** 10.3390/nu8080510

**Published:** 2016-08-19

**Authors:** Daniel Gahreman, Mehrdad Heydari, Yati Boutcher, Judith Freund, Stephen Boutcher

**Affiliations:** 1 Department of Exercise and Sport Science, Charles Darwin University, Ellengowan Drive, Casuarina 0811, Northern Territory, Australia; daniel.gahreman@cdu.edu.au; 2 Faculty of Health, University of Technology Sydney, 15 Broadway, Ultimo 2007, New South Wales, Australia; mehrdad.heydari@uts.edu.au; 3 School of Medical Sciences, Faculty of Medicine, University of New South Wales, High Street, Randwick, Sydney 2052, New South Wales, Australia; y.boutcher@unsw.edu.au (Y.B.); j.freund@unsw.edu.au (J.F.)

**Keywords:** sprinting, green tea, abdominal fat, overweight males

## Abstract

The combined effect of green tea ingestion and interval sprinting exercise on body and abdominal fat of overweight males was investigated. Participants were randomly assigned into control (C), green tea (GT), interval sprinting exercise (ISE), and green tea and ISE (GT + ISE) groups. The GT, GT + ISE, and C groups consumed three GT capsules daily. The ISE and GT + ISE groups completed 36 ISE sessions over 12 weeks. Forty eight overweight males with a mean BMI of 28.5 ± 0.92 kg/m^2^ and age of 26 ± 0.7 years acted as participants. There was a significant reduction in total and abdominal fat mass for the ISE and GT + ISE groups, *p* < 0.05, however, total and abdominal fat mass did not significantly change in the GT and C groups. There was a significant increase in total lean mass, *p* < 0.05, after the intervention for the ISE and GT + ISE groups only. There was a significant increase in fat oxidation during submaximal aerobic exercise, *p* < 0.05, after the intervention for the ISE, GT + ISE, and GT groups with no change for the C group. Following the 12-week intervention the ISE and GT + ISE groups, compared to C, recorded a significantly greater decrease in body and abdominal fat, and a significant increase in total lean mass. Ingestion of green tea by itself, however, did not result in a significant decrease in body or abdominal fat, but increased fat utilization during submaximal exercise. The combination of 12 weeks of GT ingestion and ISE did not result in greater total and abdominal fat reduction compared to 12 weeks of ISE alone.

## 1. Introduction

Aerobic exercise and dieting have been the major strategies used to combat the increasing worldwide prevalence of obesity [1]. Aerobic exercise programs have typically consisted of steady state, long duration, moderate intensity exercise (walking, jogging, swimming) for at least 40 min per session. Unfortunately, numerous studies have shown that aerobic exercise does not result in significant total body fat loss unless high volumes of exercise are performed [2]. Severe dieting has been found to result in some fat loss in the short term but of those who lose body fat over 90% will put the fat back on within five years [3]. Additionally, there are a number of unhealthy consequences that accompany severe dieting, such as a significant decrease in skeletal muscle mass [4]. This lack of success has generated interest into other fat loss strategies, such as green tea (GT) ingestion [5] and participation in interval sprinting exercise (ISE) [6].

In some studies regular GT ingestion has been shown to result in a significant decrease in body fat [7,8,9] whereas others have found no change [10,11,12]. It has been suggested that the fat loss effect of GT ingestion is brought about via a bioactive catechin called epigallocatechin gallate (EGCG) [13] which has been shown to increase fat oxidation and daily energy expenditure [14]. Habitual tea drinkers showed a 20% reduction in body fat compared to non-habitual tea drinkers [7]. Additionally, visceral [7] and abdominal fat [8] was decreased after 12 weeks of GT extract consumption.

ISE is an effective [15], enjoyable [16], and time efficient method of exercise training [6]. Tjønna et al. [15] showed that 16 weeks of ISE resulted in a significant reduction in fasting glucose, weight, and waist circumference, and a significant increase in insulin sensitivity, HDL, and maximal oxygen uptake ($\dot{V}O_{2\text{max}}$). Additionally, Trapp et al. [17] compared steady-state cycle exercise and ISE in women who performed a bout of ISE exercise lasting 8 s with a 12 s easy pedaling recovery (20 min total) for three times per week for 15 weeks. Fat mass decreased, whereas lean mass significantly increased in the ISE condition compared to no change in subcutaneous fat or lean mass after steady state exercise. An ISE protocol lasting 12 weeks with overweight males also resulted in significant decreases in total and visceral fat and significant increases in lean mass [18] and with overweight females a similar ISE program brought about significant reductions in abdominal fat and insulin resistance [19].

An important aspect of ISE may be its ability to increase fat oxidation after exercise [6]. For example, Gahreman et al. [20,21] examined the acute effect of GT extract and ISE on fat oxidation of females and males. Results indicated that participants who ingested GT before a bout of ISE significantly increased their post-exercise fat oxidation compared to an ISE alone condition. The long term effect of the combination of ISE and GT extract on body composition, aerobic fitness, and other health variables of overweight adults, however, does not appear to have been examined. Therefore, the aim of this study was to examine the effect of an ISE intervention and GT ingestion on the body composition of overweight males. It was hypothesized that the combination of 12 weeks of GT ingestion and ISE would result in greater body and abdominal fat reduction compared to 12 weeks of ISE or GT alone.

## 2. Participants and Methods

### 2.1. Participants

Forty eight healthy volunteer, non-smoking, males aged 26.1 + 0.7 years participated in the study. Regular GT consumers and individuals with known or suspected injury, cardiovascular, or musculoskeletal problems were eliminated. Participants read and signed an informed consent form which explained potential risks of DEXA, GT capsules, and blood sampling. The study was approved by the University of New South Wales Human Ethics Committee and has been registered as a clinical trial (Australian New Zealand Clinical Trials Registry, ACTRN126000096500). Project identification code was HREC10204 and date of approval was 23 November 2008. Body composition was assessed at St Vincent’s Hospital, whereas all other variables where collected and analyzed at the University of New South Wales.

### 2.2. Procedures

Forty eight participants were randomly assigned into one of four groups: interval sprinting exercise (ISE), green tea (GT), green tea and interval sprinting exercise (GT + ISE), and control (C). Our prior research using a similar ISE program with males [18] examining aerobic fitness has produced a large effect size of 2.50 (Cohen’s *d*). A recommended power is 0.8. Thus, based on a moderately large effect size, sample sizes of 6–8 males per group would provide a statistical power of 0.8 at an alpha of *p* < 0.05. Enrolment and assignment was carried out by the first author and involved picking a paper marked “C, ISE, GT, or GT + ISE” from a hat. The majority of participants were university students. The study was initiated at the start of 2010 and was completed by the end of 2011. For various reasons five participants withdrew from the study: two left the university, two relocated from the area, and one dropped out of exercise training because he needed more time to prepare for impending examinations. These non-adherents were replaced with new participants who were allocated to groups by the same method. At pre-test there was no significant difference on any measured variable between the non-adherents and those males who stayed in the study. The GT and C groups were asked to consume three GT extract or placebo capsules daily, one with each meal, whereas the GT + ISE group participated in a supervised ISE training session, three times per week. The GT + ISE group were also asked to ingest one GT capsule one hour before fasted ISE training and one capsule with lunch and dinner. On non-exercise days they were asked to ingest one GT capsule with breakfast, lunch, and dinner. Participants completed a daily GT/placebo intake capsule form. The form recorded the ingestion of capsules before each exercise session and during each daily meal. Participants were regularly reminded to fill in the form which was completed for all days of the intervention by all participants. All participants reported full compliance. The GT capsule (GNC, Pasadena, California, USA) contained 250 mg of *Camellia sinensis* extract (187.5 mg polyphenols, 125 mg EGCG, 20 mg caffeine) [22]. $\dot{V}O_{2\text{max}}$ tests for all participants were carried out before and at the end of the 12-week intervention using a TrueMax 2400 Metabolic Cart (ParvoMedics Inc., East Sandy, UT, USA) and a cycle ergometer (Ergomedic 839E, Monark, Vansbro, Sweden). The protocol started with a 3-min warm-up at 30 watts (W) and then increased by 1 W every two seconds. Participants were asked to maintain a pedal rate at 60 to 70 revolutions per minute (RPM) throughout the test. Power output continued to increase until participants failed to maintain pedal frequency despite verbal encouragement. Participants were asked to give a rating of perceived exertion (RPE) every minute throughout the test [23]. Heart rate was recorded using a Polar S810I telemetry system (Polar, Kempele, Finland).

### 2.3. Body Composition

Standard stadiometer and bioimpedance (Tanita, Itabashi-ku, Tokyo, Japan) were used to measure height and body weight. A dual energy X-ray absorptiometry (DEXA) scan with a Lunar Prodigy scanner (software version 7.51, GE Corporation, New York, NY, USA) was used to measure body mass and percentage body fat. Fat mass, along with lean mass, in kg were measured for the whole body. DEXA also provided information on abdominal fat as an indicator of central adiposity. Body-mass index (BMI) was calculated by dividing weight by height squared (kg/m^2^).

### 2.4. Interval Sprinting Exercise Protocol

Participants in the exercise groups completed 36 fully-supervised ISE sessions over 12 weeks. A typical exercise session consisted of a 5-min warm-up, 20 min of ISE, and a 5-min cool-down. The main exercise session consisted of 60 bouts of 8 s of high-intensity cycling followed by 12 s of slow cycling recovery. The ISE workload was set at 85% to 90% of each participant’s maximum heart rate at a cadence between 100 and 120 RPM and recovery was set at the same amount of resistance but at a cadence of 50 to 60 RPM. Participants were instructed to keep their intensity at a level to ensure the average heart rate for exercise fell within their individual maximal heart rate range and intensity was increased when heart rate during ISE fell below maximal heart rate range. ISE was coordinated with a pre-recorded compact disk counting down each sprint in a 3-2-1 manner. Training cycling data included continuous recording of heart rate and RPM, whereas RPE was assessed at 5-min intervals.

### 2.5. Assays

Blood samples were taken from an antecubital vein after overnight fasting prior to the start of the trial and at the end of the 12-week intervention. Whole blood in EDTA tubes was spun immediately in a chilled centrifuge (Model Megafuge 1.0R, Heraeus, Germany) at 4 °C at 3500 RPM for 10 min and plasma was stored immediately at −86 °C for later analysis. Blood lipid profiles including triglyceride (TG), low-density lipoprotein cholesterol (LDL-C), high-density lipoprotein cholesterol (HDL-C), and glucose were evaluated at baseline and at the end of the 12-week trial by automated enzymatic methods (Cholestech LDX, Hayward, CA, USA) from whole blood.

### 2.6. Diet

Participants were requested to adhere to their normal diet during the intervention. They were also asked to record their diet for three consecutive days before and after completing the intervention. Diet was analyzed using SERVE computer software (Professional edition, version 5.1, SERVE Nutrition Management Systems, Sydney, New South Wales, Australia). Forty eight hours before the final testing session, participants were asked to stop ingesting GT capsules.

### 2.7. Statistical Analysis

Data were analyzed with the Statistical Package for Social Science for Windows software (SPSS Version 22, Armonk, New York, NY, USA). To examine changes after the intervention an analysis of covariance (ANCOVA) was used to evaluate differences between the groups for variables that did not violate ANCOVA assumptions. Where assumptions were violated one way ANOVA was conducted on the difference scores. Effect sizes were calculated using Cohen’s *d* with values of 0.0 to 0.2 considered a small, above 0.2 to 0.5 considered a medium, and above 0.5 considered a large effect. The statistical analysis was considered significant when the probability level was less than 0.05.

## 3. Results

There was no significant difference between any group for body mass, BMI, and $\dot{V}O_{2\text{max}}$ before the training program (Table 1).

### 3.1. Exercise Training Heart Rate, RPE, and Power Output

The average heart rate during the 36 ISE training sessions for the ISE group was 161 ± 2.3 beats per min (bpm) which corresponded to 85% of maximal heart rate and for the GT + ISE group was 165 ± 2.4 bpm which corresponded to 86% of maximal heart rate. Average RPE was 13.7 ± 0.6 for the ISE group and 13.6 ± 0.6 for the GT+ISE group. Maximal power output significantly increased after the intervention, *p* < 0.001, in the ISE and GT+ISE groups by 41 and 40 watts. The mean pedal RPM during the 8 s sprint was 110 ± 2.3 and during the recovery period was 57 ± 1.5.

### 3.2. Aerobic Power

There was a significant increase in relative $\dot{V}O_{2\text{max}}$ after the intervention for the GT + ISE, *p* < 0.05, *d* = 2.85, and ISE groups, *p* < 0.05, *d* = 4.58, but no significant change was found for the GT or C groups. Relative $\dot{V}O_{2\text{max}}$ increased by 18% for the GT + ISE group and 15% for the ISE group (Table 1).

### 3.3. BMI and Waist Circumference

There was a significant decrease in BMI after the intervention for the GT + ISE (*p* < 0.05, *d* = 0.44) and ISE (*p* < 0.05, *d* = 0.70) groups, but BMI did not show any significant change in the GT or C groups (Table 1). Waist circumference was significantly reduced, *p* < 0.05, *d* = 0.97, by 3.3 cm in the GT + ISE and by 4.4 cm in the ISE group, *p* < 0.05, *d* = 1.92, compared to baseline (Table 1), whereas no change occurred in the GT and C groups. At week 6, waist circumference was significantly reduced, *p* < 0.05, by 2.1 cm for the GT + ISE and 2.9 cm for the ISE group, whereas no change occurred for the GT or C groups.

### 3.4. Body Composition

**Figure 1:**
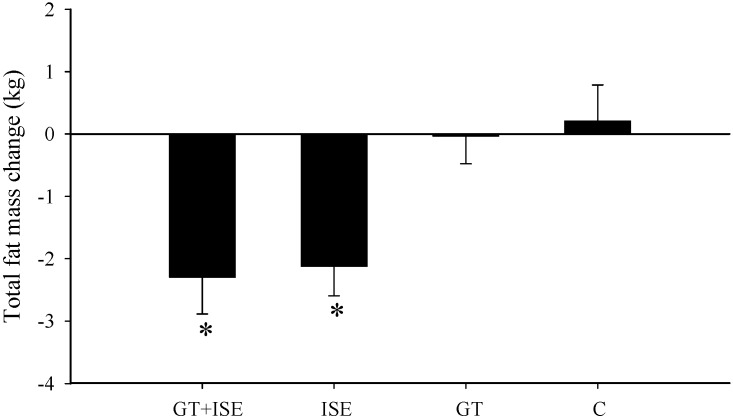
Change in total body fat mass after the 12-week intervention for the control (C), interval sprinting exercise (ISE), green tea (GT), and green tea and interval sprinting exercise (GT + ISE) groups. * Significantly different from the control group, *p* < 0.05.

There was a significant decrease in total body mass after the 12-week intervention for the GT + ISE (*p* < 0.05, *d* = 0.31) and ISE (*p* < 0.05, *d* = 0.43) groups (Table 2). There was also a significant decrease in total fat mass after the intervention for the GT+ISE (*p* < 0.05, *d* = 0.65) and ISE (*p* < 0.05, *d* = 0.83) groups, by 8% and 7% (Table 2), however, total fat mass showed no significant change in the GT or C groups (Figure 1).

**Figure 2:**
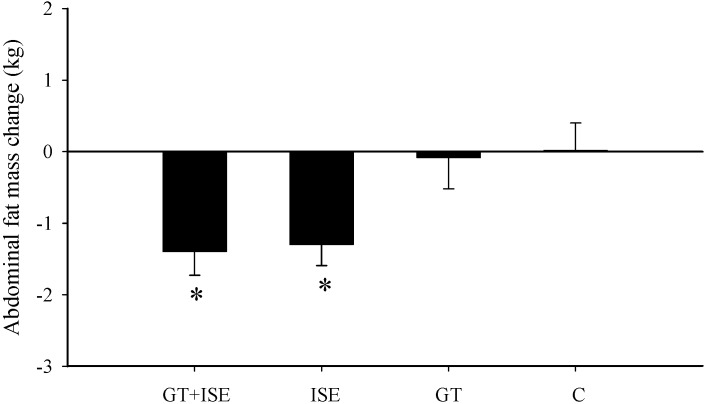
Change in abdominal fat mass after the 12-week intervention for the control (C), interval sprinting exercise (ISE), green tea (GT), and green tea and interval sprinting exercise (GT + ISE) groups. * Significantly different from the control group, *p* < 0.05.

There was a significant decrease in abdominal fat mass after the intervention for the GT + ISE (*p* < 0.05, *d* = 0.92) and ISE groups (*p* < 0.05, *d* = 0.79) by 10% and 6% with no change for the GT and C groups (Figure 2). There was a significant increase in total lean mass for the GT + ISE (*p* < 0.05, *d* = 0.23, +0.57 kg) and ISE (*p* < 0.05, *d* = 0.26, +0.56 kg) groups after the intervention with no significant change for the other two groups (Table 2). Lean mass was increased mostly in the legs and trunk (Table 2).

### 3.5. Blood Lipids

There was a significant decrease in total cholesterol after the intervention for the GT+ISE group, *p* < 0.05, *d* = 1.15, but no change was found for the GT, ISE, or C groups, *p* > 0.05 (Table 1). Also a significant decrease in TG after the intervention for the ISE group was found, *p* < 0.05, *d* = 1.90, but no change for the GT, GT+ISE, or C groups existed, *p* > 0.05 (Table 1). 

### 3.6. Diet 

There was no significant change in diet for any group before or after the 12-week intervention. Diet for all groups was similar and total energy, averaged across groups before and after the intervention, was 8290 + 386 kJ.day^-1^ that comprised of 45.5% carbohydrate, 18.9% protein, and 34.8% fat (Table 3). 

### 3.7. RER and Heart Rate during Submaximal Aerobic Exercise

Compared to the C group the GT + ISE (*p* < 0.05, *d* = 3.50, 15%), the ISE (*p* < 0.05, *d* = 267, 14%), and the GT group (*p* < 0.05, *d* = 1.00, 8%) recorded a significant decrease in RER during all stages of the $\dot{V}O_{2\text{max}}$ test after 12 weeks (Table 4). Compared to the C group the GT + ISE (*p* < 0.05, *d* = 3.03) and ISE (*p* < 0.05, *d* = 2.30) groups also recorded a significant decrease in heart rate during all stages of the $\dot{V}O_{2\text{max}}$ test after 12 weeks of ISE (Table 4). The GT group also recorded lower heart rates at every stage during exercise although the decrease was not significant, *p* > 0.05, Table 4.

## 4. Discussion

Following the 12-week intervention overweight males in both exercise groups (GT + ISE and ISE) recorded a significant increase in $\dot{V}O_{2\text{max}}$ and a significant decrease in total body fat mass, abdominal fat mass, and waist circumference. The GT + ISE and the ISE groups, compared to the C group, also significantly increased lean mass. The GT + ISE, ISE, and GT groups, compared to the C group, also recorded a significant increase in fat oxidation during the submaximal stages of the post $\dot{V}O_{2\text{max}}$ test. Ingestion of GT did not result in a significant decrease in body or abdominal fat but resulted in increased fat utilization during submaximal exercise.

There was a significant increase in $\dot{V}O_{2\text{max}}$ for the GT+ISE and ISE groups which was 18% and 15% following the 12 weeks or 12 h of ISE. Trapp et al. [17] used the same ISE training protocol for 15 weeks and reported a 24% increase in $\dot{V}O_{2\text{max}}$, whereas Heydari et al. [18] exercised overweight males for 12 weeks and found a 15% increase in $\dot{V}O_{2\text{max}}$. Talanian et al. [24] found that two weeks of ISE significantly elevated aerobic power and also resulted in enhanced mitochondrial capacity. The oxidative enzyme β-hydroxy-acyl-CoA dehydrogenase was used as a marker of mitochondrial volume. Additionally, stroke volume at rest and during aerobic exercise has been found to increase after ISE training [25,26,27]. These central and peripheral adaptations are likely to make a major contribution to the consistent increase in $\dot{V}O_{2\text{max}}$ found after ISE training [28]. As low aerobic power has been shown to be a predictor of mortality and morbidity [29] future studies are needed to assess the clinical benefits of increasing aerobic power through participation in ISE.

With regard to body fat, these results support prior research that has shown that ISE, compared to steady state aerobic exercise, is more effective at reducing total body fat after a much lower exercise volume. Trapp et al. [17] reported a 2.6 kg significant reduction in body fat of females after 15 weeks or 15 h of ISE training compared to no change in a steady state cycle group. Additionally, 12 weeks of ISE resulted in a 2.5 kg and 2.0 kg decrease in total body fat of overweight women [19] and men [18]. In the current study, after 12 weeks or 12 h of ISE, the average body fat loss in the ISE group was 2.0 kg. Continuous walking and jogging for at least 45 min per session for at least three times per week has resulted in only small decreases in total body fat [2], therefore, participation in ISE appears to be a time sparing option for those people interested in losing subcutaneous fat.

The significant decrease in abdominal fat for the ISE group supports the results of previous ISE studies [17,18,19] and may indicate that participants also experienced a loss in visceral fat as waist circumference was significantly decreased by 4.3 cm in the ISE group. It has previously been found that the waist circumference change after ISE of young males was significantly correlated to visceral fat change, measured by computed tomography [18], therefore, the decrease in waist circumference found in the present study may indicate that visceral fat was also reduced. Compared to overall obesity, visceral fat is a much stronger predictor of cardiovascular disease risk [30]. Thus, the ability of ISE to reduce visceral fat quickly may have implications for metabolic health. For example, visceral fat reduction was correlated with improvement in glucose and lipid metabolism [31] and reduced risk for atherosclerotic disease [32]. That waist circumference was significantly reduced by just under 3 cm after six weeks or 6 h of ISE also suggests that a minimal amount of interval sprinting may result in a decrease in visceral fat.

Participants in the GT + ISE and ISE groups significantly increased their lean mass. It has been previously shown that leg and trunk lean mass was significantly increased in young females by 0.6 kg after 15 weeks of ISE [17], whereas Boudou et al. [33] using MRI found a significant increase in thigh muscle cross-sectional area of older males and females after ISE training. Heydari et al. [18] also found that leg and trunk lean mass was significantly increased in overweight males by 1.2 kg after 12 weeks of ISE. The 0.6 kg increase in total lean mass found after ISE in the present study confirms the ability of this type of exercise to increase lean mass. The increase in lean mass after ISE is important for weight control as muscle mass typically decreases after dietary restriction [4] and remains unchanged after aerobic exercise training [34].

Although GT ingestion has been suggested to be an anti-obesity agent and has been shown to reduce total body fat [7,8] the present study did not find a significant reduction in total body fat after 12 weeks of GT consumption. These results are in agreement with previous studies which did not find any significant change in total body fat and waist circumference after GT extract consumption [10,11,12]. Hsu et al. [10] reported a 0.3% reduction in total body mass equivalent to 150 g over a three-month study. In the current study a 3% reduction in total body fat (0.55 kg) after 12 weeks of GT consumption was found. Thus, it appears that 12 weeks of GT, with a daily dose of 375 EGCG, does not result in a significant reduction in fat mass. That participants did not gain any total fat, abdominal fat, or increase their waist circumference, however, may be important as it is feasible that GT may prevent fat gain associated with sedentary living. The results of animal [35] and human [11] studies support this notion. For example, Wolfram et al. [36] fed a high fat diet and EGCG to rodents and showed obesity-preventing effects of EGCG. Rodents, who were exposed to a high-fat diet plus EGCG, gained less body weight compared to a high-fat diet without EGCG supplementation.

The proportion of whole-body fat utilization during exercise, assessed by RER, during the submaximal stages of the pre and post-intervention $\dot{V}O_{2\text{max}}$ tests increased after 12 weeks of ISE by 15% and 14% in the GT + ISE and ISE groups, and by 8% in the GT alone group. These findings suggest that the capacity to utilize more fat during moderate intensity aerobic exercise is enhanced with ISE training. That green tea ingestion by itself increased fat oxidation during aerobic exercise supports prior research which has shown that 10 weeks of aerobic training with green tea ingestion increased the proportion of whole-body fat utilization during exercise [37]. Additionally, a four-week GT intervention by itself favorably enhanced fat substrate utilization during a steady-state bout of exercise [38]. Interestingly, post-intervention heart rates during the $\dot{V}O_{2\text{max}}$ test for the GT group, although not reaching significance, were lower at every submaximal workload compared to pre-test (Table 4). The mechanism underlying this response is undetermined.

With regard to blood lipids Hsu et al. [10] reported a significant reduction in TG and LDL and a significant increase in HDL following 12 weeks of green tea consumption in overweight women. Participants in the present study possessed lower resting levels of TG, LDL, and glucose and higher levels of HDL. Furthermore, these participants were younger and, although overweight, had normal blood lipid levels, therefore, blood lipids and glucose were unlikely to change. No significant differences in glucose, HDL, LDL, or insulin were found after GT consumption although TC and TG did decrease significantly. Thus, GT may have a more positive effect on lipid profiles for individuals who are hyperlipidemic.

Another factor that may influence the effect of GT on body fat is dosage. It has been shown that plasma EGCG concentration reached ~0.87 μM and ~0.32 μM in 4 h after 800 mg and 600 mg of EGCG consumption [39]. The biological effects of EGCG have been observed in vitro when tea catechin concentration reached 10 to 100 μM. It was found that blood EGCG concentration was detected in only five out of 41 participants who consumed 302 mg of EGCG [40]. In the current study participants consumed 375 mg of EGCG which may be below the threshold for a significant biological response. The dose that participants received, however, was equivalent to about nine cups of GT per day which is considered to be a level that does not bring about gastrointestinal problems [41]. Although the GT+ISE group ingested one of their daily GT capsules in a fasted state before exercising they did not lose greater body fat compared to the ISE group. GT ingestion before one bout of ISE increased post-exercise fat oxidation by 29% in untrained females [20] and by 17% in untrained males [21]. However, post-exercise fat oxidation rate in these studies was assessed in the fasted state. In the current study participants, having finished exercise, consumed their normal breakfast. Consequently, post-exercise fat oxidation may have been blunted by carbohydrate ingestion. Therefore, more studies need to be conducted examining the impact of GT ingestion before ISE followed by a post-exercise fasted state on body composition change. Additionally, the total body fat of the GT group was less than the other groups which may have contributed to the lack of a GT-induced fat loss effect.

This study is relatively small and the intervention lasted 12 weeks. We did not measure physical activity outside the study and diet was only assessed before and after the intervention, whereas ingestion of daily green tea was monitored by self-report. Thus, lack of dietary control could have influenced study outcomes. In addition, not assessing blood levels of EGCG and other GT catechins prevented gaining information about responders and non-responders. Another limitation is that these results can only be generalized to young overweight males. The strength of the study was the strict training program where every session was monitored allowing an examination of the effects of the intervention on aerobic fitness and body composition.

## 5. Conclusions

In conclusion, it was found that after 12 weeks or 12 h of ISE, overweight males recorded significantly lower body fat and greater $\dot{V}O_{2\text{max}}$ in the interval sprinting exercise only and interval sprinting exercise and green tea groups. Both exercise groups also recorded a significant increase in lean mass. Ingestion of green tea, by itself, did not decrease body or abdominal fat.

## Figures and Tables

**Table 1:** Weight, BMI, waist circumference, maximal oxygen uptake, and lipid levels of the green tea plus interval sprinting exercise (GT + ISE), interval sprinting exercise (ISE), green tea (GT), and control (C) groups before and after the 12-week intervention (mean and standard error).

Variable	GT + ISE		ISE		GT		C	
	Pre	Post	Pre	Post	Pre	Post	Pre	Post
Weight	88.48 ± 5.57	86.79 ± 5.44 *	88.08 ± 3.84	86.43 ± 3.75 *	85.83 ± 1.95	85.72 ± 2	91.19 ± 4.03	91.87 ± 4.17
BMI	29.04 ± 1.28	28.48 ± 1.26 *	28.67 ± 0.78	28.13 ± 0.77 *	26.95 ± 0.29	26.92 ± 0.32	29.51 ± 1.31	29.71 ± 1.3
Waist circumference	96.17 ± 3.4	92.88 ± 3.38 *	94.25 ± 2.17	89.88 ± 2.33 *	92.42 ± 1.15	92.46 ± 1.59	95.08 ± 2.75	96.07 ± 2.88
$\dot{V}O_{2\text{max}}$ (mL/kg/min)	34.23 ± 2.11	40.38 ± 2.21 *	34.08 ± 1.35	39.32 ± 0.94 *	39.77 ± 2.26	40.39 ± 1.68	27.68 ± 1.34	28.19 ± 1.40
Cholesterol (mmol/L)	4.61 ± 0.36	4.23 ± 0.3 *	4.2 ± 0.28	3.92 ± 0.26	4.61 ± 0.3	4.57 ± 0.26	4.38 ± 0.27	4.38 ± 0.26
HDL (mmol/L)	1.06 ± 0.12	1.02 ± 0.09	1.34 ± 0.12	1.35 ± 0.11	1.15 ± 0.09	1.17 ± 0.09	0.98 ± 0.09	0.97 ± 0.1
Triglycerides (mmol/L)	1.42 ± 0.23	1.21 ± 0.15	1.0 ± 0.11	0.79 ± 0.11 *	0.98 ± 0.17	0.99 ± 0.19	1.39 ± 0.19	1.38 ± 0.23
LDL (mmol/L)	2.9 ± 0.3	2.87 ± 0.35	2.42 ± 0.19	2.25 ± 0.18	3.03 ± 0.22	2.95 ± 0.23	2.76 ± 0.25	2.74 ± 0.24
TC/HDL (mmol/L)	4.73 ± 0.53	4.46 ± 0.45	3.29 ± 0.23	3 ± 0.16	4.15 ± 0.35	4 ± 0.41	4.76 ± 0.39	4.73 ± 0.36
Glucose (mmol/L)	5.06 ± 0.08	4.96 ± 0.13	4.74 ± 0.21	4.81 ± 0.15	4.8 ± 0.13	4.84 ± 0.13	4.71 ± 0.15	4.73 ± 0.12

* Pre-post change significantly different from that of the control group, *p* < 0.05.

**Table 2:** Total body and abdominal fat and lean mass of the green tea plus interval sprinting exercise (GT + ISE), interval sprinting exercise (ISE), green tea (GT), and control (C) groups before and after the 12-week intervention (mean and standard error).

Variable	GT + ISE		ISE		GT		C	
	Pre	Post	Pre	Post	Pre	Post	Pre	Post
Total body fat mass (kg)	28.47 ± 3.6	26.19 ± 3.5 *	29.63 ± 2.3	27.68 ± 2.3 *	21.48 ± 1.3	20.93 ± 1.7	33.06 ± 3.2	33.04 ± 3.4
Abdominal fat mass (kg)	2.31 ± 0.3	2.07 ± 0.2 *	2.33 ± 0.2	2.2 ± 0.2 *	1.66 ± 0.1	1.61 ± 0.2	2.56 ± 0.3	2.57 ± 0.3
Arm lean mass (kg)	6.98 ± 0.4	7.17 ± 0.4	6.96 ± 0.4	6.91 ± 0.4	8.33 ± 0.4	8.37 ± 0.4	6.80 ± 0.4	6.86 ± 0.4
Trunk lean mass (kg)	26.35 ± 1.1	26.55 ± 1.2	25.63 ± 1.0	25.920.9	28.11 ± 1.0	28.16 ± 1.0	25.63 ± 0.1	25.69 ± 1.0
Leg lean mass (kg)	20.3 ± 1.0	20.52 ± 0.9	18.97 ± 0.9	19.28 ± 0.8	21.44 ± 0.7	21.35 ± 0.7	19.47 ± 0.1	19.4 ± 0.6
Total lean mass (kg)	58.13 ± 2.4	58.7 ± 2.4 *	55.76 ± 2.3	56.32 ± 2.0 *	62.32 ± 1.9	62.31 ± 2.0	55.72 ± 1.7	55.67 ± 1.6

* Pre-post change significantly different from that of the control group, *p* < 0.05.

**Table 3:** Diet of the green tea plus interval sprinting exercise (GT + ISE), interval sprinting exercise (ISE), green tea (GT), and control (C) groups before and after the 12-week intervention (mean and standard error).

Variable	GT + ISE		ISE		GT		C	
	Pre	Post	Pre	Post	Pre	Post	Pre	Post
Total energy intake (kJ·day^-1^)	8655 ± 528	8934 ± 485	7703 ± 260	7753 ± 244	8235 ± 412	8572 ± 436	8568 ± 342	8641 ± 343
% carbohydrates	43.42 ± 2.5	41.31 ± 2.1	43.11 ± 2.1	43.33 ± 2.0	49.16 ± 1.4	45.99 ± 1.6	46.18 ± 1.8	46.78 ± 2.0
% protein	18.27 ± 0.8	18.52 ± 0.7	19.45 ± 1.3	18.93 ± 1.3	19.61 ± 1.2	20.31 ± 1.1	18.18 ± 1.1	18.33 ± 1.5
% fat	35.22 ± 2.5	35.88 ± 2.1	37.28 ± 2.0	37.58 ± 1.8	31.01 ± 1.4	33.22 ± 1.5	35.66 ± 1.7	34.89 ± 1.4
% saturated fat	12.46 ± 1.1	12.57 ± 1.1	13.71 ± 0.9	13.11 ± 0.9	11.21 ± 0.7	11.02 ± 0.7	12.40 ± 0.7	11.29 ± 0.6
% mono-unsaturated fat	13.28 ± 0.97	13.76 ± 0.78	14.01 ± 0.82	14.27 ± 0.85	11.40 ± 0.51	12.96 ± 0.68	13.58 ± 0.68	13.84 ± 0.70
% poly- unsaturated fat	6.46 ± 0.60	6.87 ± 0.65	6.23 ± 0.59	6.91 ± 0.77	5.11 ± 0.65	6.16 ± 0.76	6.26 ± 0.61	6.50 ± 0.7
Cholesterol (mg)	356.24 ± 40	402.66 ± 36	337.68 ± 21	312.87 ± 25	406.98 ± 63	450.28 ± 43	304.83 ± 41	381.80 ± 56
Fibre (g)	21.75 ± 3.0	25.58 ± 2.8	16.78 ± 2.1	19.44 ± 2.3	19.43 ± 2.6	19.07 ± 3.1	20.28 ± 2.4	21.39 ± 2.8
Sodium (mg)	3039 ± 490	3269 ± 449	2379 ± 399	2375 ± 398	3121 ± 480	3323 ± 472	2389 ± 345	2546 ± 334

**Table 4:** Respiratory exchange ratio (RER) and heart rate (HR) during submaximal exercise before and after the intervention for the control, interval sprinting exercise, green tea, and green tea and interval sprinting exercise groups (mean and SEM).

Group	Variable		Rest	Submaximal Exercise (W)						
				60 W	90 W	120 W	150 W	180 W	210 W	240 W
**Exercise**	RER	Pre	0.85 ± 0.01	0.88 ± 0.03	0.90 ± 0.03	0.92 ± 0.03	0.99 ± 0.03	1.07 ± 0.03	1.14 ± 0.04	1.21 ±0.04
		Post	0.86 ± 0.01	0.85 ± 0.02	0.85 ± 0.02	0.87 ± 0.03	0.91 ± 0.03	0.98 ± 0.03	1.04 ± 0.03	1.10 ± 0.04 *
	HR	Pre	63 ± 3	98 ± 3.1	104 ± 3.2	115 ± 3.4	127 ± 4.2	139 ± 4.9	153 ± 4.8	166 ± 4.1
		Post	58 ± 2	93 ± 3.5	97 ± 3.0	108 ± 3.5	119 ± 3.9	129 ± 4.2	141 ± 4.4	153 ± 4.4 *
**Exercise/green tea**	RER	Pre	0.85 ± 0.02	0.84 ± 0.01	0.86 ± 0.02	0.92 ± 0.02	1.00 ± 0.02	1.10 ± 0.02	1.15 ± 0.03	1.22 ± 0.03
		Post	0.83 ± 0.02	0.83 ± 0.01	0.82 ± 0.02	0.86 ± 0.01	0.92 ± 0.02	0.99 ± 0.02	1.05 ± 0.02	1.10 ± 0.04 *
	HR	Pre	62 ± 2	100 ± 3.5	109 ± 3.2	119 ± 3.0	130 ± 3.0	143 ± 3.4	156 ± 4.1	168 ± 4.1
		Post	58 ± 1	94 ± 3.4	100 ± 2.8	108 ± 2.5	119 ± 2.4	133 ± 3.3	144 ± 3.3	157 ± 4.0 *
**Green tea**	RER	Pre	0.85 ± 0.02	0.84 ± 0.02	0.84 ± 0.02	0.88± 0.02	0.94± 0.02	1.01 ± 0.02	1.08 ± 0.03	1.15 ± 0.03
		Post	0.83 ± 0.01	0.82 ± 0.02	0.83 ± 0.02	0.85 ± 0.02	0.90 ± 0.02	0.98 ± 0.03	1.06 ± 0.03	1.14 ± 0.04 *
	HR	Pre	62 ± 3	93 ± 2.3	100 ± 2.5	109 ± 3.1	120 ± 3.6	132 ± 4.3	145 ± 4.6	157 ± 4.8
		Post	61 ± 3	91 ± 3.2	97 ± 3.4	107 ± 4.0	118 ± 4.6	129 ± 5.5	142 ± 5.5	154 ± 5.5
**Control**	RER	Pre	0.85 ± 0.02	0.85 ± 0.02	0.85 ± 0.02	0.91 ± 0.01	1.01 ± 0.02	1.10 ± 0.02	1.18 ± 0.02	1.24 ± 0.03
		Post	0.87 ± 0.02	0.84 ± 0.01	0.84 ± 0.02	0.91 ± 0.02	1.01 ± 0.02	1.09 ± 0.02	1.16 ± 0.02	1.22 ± 0.03
	HR	Pre	63 ± 2	105 ± 2.5	112 ± 3.0	123 ± 3.4	136 ± 4.0	148 ± 4.1	161 ± 3.8	169 ± 3.4
		Post	65 ± 2	103 ± 2.2	112 ± 2.1	122 ± 2.7	134 ± 3.0	146 ± 3.3	158 ± 3.2	168 ± 2.6

* Decrease after exercise significantly greater than that of the control group, *p* < 0.05; W = watts.
